# Nuclear respiratory factor 1 promotes the growth of liver hepatocellular carcinoma cells via E2F1 transcriptional activation

**DOI:** 10.1186/s12876-022-02260-7

**Published:** 2022-04-21

**Authors:** Dan Wang, Baolan Wan, Xiaojing Zhang, Pingping Sun, Shu Lu, Chenxu Liu, Li Zhu

**Affiliations:** 1 Institute of Special Environmental Medicine, Nantong University, 9 Se Yuan Road, Nantong, 226019 Jiangsu China; 2 Department of Clinical Biobank, Affiliated Hospital of Nantong University, Nantong, 226001 Jiangsu China; 3 Department of Intensive Care Unit, Affiliated Hospital of Nantong University, Nantong, 226001 Jiangsu China; 4 Department of Biochemistry and Molecular Biology, Medical School, Nantong University, Nantong, 226001 Jiangsu China

**Keywords:** Hepatocellular carcinoma, Prognosis, Cell proliferation, Gene expression regulation

## Abstract

**Background:**

Recent studies have shown that functional mitochondria are essential for cancer cells. Nuclear respiratory factor 1 (NRF1) is a transcription factor that activates mitochondrial biogenesis and the expression of the respiratory chain, but little is known about its role and underlying mechanism in liver hepatocellular carcinoma (LIHC).

**Methods:**

NRF1 expression was analyzed via public databases and 24 paired LIHC samples. Clinical-pathological information and follow-up data were collected from 165 patients with LIHC or online datasets. Furthermore, cellular proliferation and the cell cycle were analyzed by MTT, Clone-forming assay and flow cytometric analyses. NRF1 target genes were analyzed by Chromatin immunoprecipitation sequencing (ChIP-Seq). PCR and WB analysis was performed to detect the expression of related genes. ChIP and luciferase activity assays were used to identify NRF1 binding sites.

**Results:**

Our results showed that NRF1 expression was upregulated in LIHC compared to normal tissues. NRF1 expression was associated with tumour size and poor prognosis in patients. Knockdown of NRF1 repressed cell proliferation and overexpression of NRF1 accelerated the G_1_/S phase transition. Additionally, data from ChIP-seq pointed out that some NRF1 target genes are involved in the cell cycle. Our findings indicated that NRF1 directly binds to the *E2F1* promoter as a transcription factor and regulates its gene expression.

**Conclusion:**

Therefore, this study revealed that NRF1 promotes cancer cell growth via the indirect transcriptional activation of E2F1 and is a potential biomarker in LIHC.

**Supplementary Information:**

The online version contains supplementary material available at 10.1186/s12876-022-02260-7.

## Background

Liver cancer is the third leading cause of cancer death worldwide [1]. Most primary liver cancer occurring worldwide is liver hepatocellular carcinoma (LIHC) [2, 3]. The early diagnosis of LIHC is complicated thus far. The overall five-year survival rate is extremely low because greater than 60% of patients are diagnosed in advanced stages [4–6]. Thus, an effective biomarker is urgently needed to estimate the prognosis.

Hepatocytes, which are rich in mitochondria, have developed diverse mechanisms to maintain mitochondrial homeostasis by regulating mitochondrial dynamics, biogenesis and degradation [7, 8]. Mitochondrial reactive oxygen species (mROS) mediate metabolic pathway signalling; alterations in these pathways affect the development and progression of chronic liver diseases and tumours [9, 10]. Paradoxically, mitochondrial metabolism can be both advantageous and detrimental to cancer metastasis and therapy resistance [10]. Recently, emerging studies have shown that functional mitochondria are essential for cancer cells [11]. Mitochondria in cancer cells are different from their normal counterparts in structure and function [12–14]. Beyond the classical role in energy and metabolic mechanisms, both mitochondrial DNA (mtDNA) defects and increased mitochondrial fission have been reported in many cancers [15]. Importantly, mitochondrial biogenesis and quality control are often upregulated in cancers and play a critical role in oncogenic signalling pathways [11, 16]. Nuclear respiratory factor 1 (NRF1) is a transcription factor known to directly regulate several nuclear-encoded electron transport chain proteins [17]. In addition, NRF1 is indirectly involved in regulating the expression of mtDNA transcription by coactivation with peroxisome proliferator-activated receptor gamma coactivator 1α (PGC-1α) [18]. Thus, NRF1 plays an essential role in mitochondrial biogenesis. Satoh et al. identified that NRF1 target genes played a pivotal role in the regulation of extramitochondrial biological processes, including DNA damage repair, protein translation initiation, and ubiquitin-mediated protein degradation [19]. NRF1 has also been identified as a valuable biomarker for breast cancer diagnosis and prognosis [20].

However, NRF1 and its target genes, whose expression pattern and biological function in tumours, are largely unknown. In this study, we aimed to investigate whether NRF1 can affect liver cancer cell growth. These findings might uncover a mechanism by which NRF1 is involved in LIHC progression.

## Materials and methods

### Evidence from the public databases

Gene expression and clinical annotation data from The Cancer Genome Atlas (TCGA) were downloaded (https://portal.gdc.cancer.gov/). The online database Tumor Immune Estimation Resource (TIMER) (https://cistrome.shinyapps.io/timer/) [21], Gene Expression Profiling Interactive Analysis (GEPIA2) (http://gepia2.cancer-pku.cn/#index) [22] and the Encyclopedia of RNA Interactomes (ENCORI) (http://starbase.sysu.edu.cn/) [23] were used to analyse the expression of IKBIP in LIHC and normal tissues. The prognosis value and associations between NRF1 expression and stage were also obtained from GEPIA2.

### Study populations

A total of 165 formalin-fixed, paraffin-embedded samples were excised from fresh LIHC surgical samples. The clinicopathological features included sex, age at diagnosis, differentiation, vascular invasion, TNM stage, tumour size and cirrhosis. None of the patients received radiotherapy, chemotherapy, or immunotherapy prior to surgery. The overall survival duration was defined as the interval from the date of the first biopsy to the date of death from disease.

### Immunohistochemistry (IHC)

LIHC tissue microarray (TMA) slides from patients were used for NRF1 staining with a Tissue Microarray System (Quick-Ray, UT06, UNITMA, Korea). Core tissue biopsies (2 mm in diameter), which were taken from individual paraffin-embedded sample sections, were arranged in new recipient paraffin blocks. IHC analysis was performed as previously described [24]. The slides were incubated with the primary antibody against NRF1 (Abcam, Cambridge, MA, USA) at 4 °C overnight. Three trained pathologists were blinded to evaluate NRF1 immunostaining. There were two estimated variables: intensity (0 to 3 as negative, weak, moderate or strong) and percentage (0% to 100%). The degree of NRF1 expression was quantified using a two-level grading system defined as follows: score ≤ 60 defined as low, otherwise defined as high.

### Cell culture, cell transfection and lentivirus infection

HepG2 cells were maintained in Dulbecco’s modified Eagle’s medium (DMEM) (HyClone, UT, USA) containing 10% foetal bovine serum (HyClone, UT, USA) and were cultured at 37 °C with 5% CO_2_ in an incubator. Cells were transiently transfected with plasmids or siRNA duplexes using Lipofectamine 2000 Transfection Reagent (Invitrogen, CA, USA) following the manufacturer's protocol. NRF1 overexpression constructs were generated into the Ubi-MCS-3FLAG-CBh-gcGFP-IRES-puromycin lentiviral vector (GeneChem, Shanghai, CHN). The lentivirus infection was manipulated according to the instructions.

### Chromatin immunoprecipitation sequencing (ChIP-Seq) dataset of NRF1 binding sites and molecular pathway analysis

ChIP was performed using the SimpleChIP® Plus Enzymatic Chromatin IP Kit (Cell Signaling Technology, MA, USA) as described in the manufacturer’s protocol. Briefly, cells were washed and fixed in 1% formaldehyde at room temperature. Then, the cells were collected and lysed to release the nuclei. Nuclei were then isolated before being subjected to micrococcal nuclease. The lysate was then immunoprecipitated with NRF1 antibodies (Abcam, MA, USA) or a negative control IgG. The pulled-down chromatin was washed, reverse-crosslinked, purified and detected by deep sequencing (Vazyme Biotech, Nanjing, China). To identify the pathways relevant to ChIP-Seq-based NRF1 target genes, we used Database for Annotation, Visualization and Integrated Discovery (DAVID) v6.8 (https://david.abcc.ncifcrf.gov/) [25], Metascape (http://metascape.org/gp/index.html#/main/step1) [26] and KOBAS (http://kobas.cbi.pku.edu.cn/) [27] to analyse the sequencing data.

### Gene silencing

Human NRF1-specific siRNA (siNRF1) duplexes were designed and synthesized by GenePharma Co., Ltd. (GenePharma, Shanghai, CHN). The siNRF1 sequences were as follows: siNRF1, 5′-CACAUUGGCUGAUGCUUCAUU-3′.

### RNA isolation and quantitative real-time PCR

RNA was isolated using TRIzol reagent (Invitrogen, CA, USA) and treated with DNase I (Promega, WI, USA) before cDNA synthesis. cDNA was synthesized by a Transcript First-Strand cDNA Synthesis Kit (Vazyme Biotech, Nanjing, China). Quantitative real-time PCR was performed using AceQ qPCR SYBR Green Master Mix (High ROX Premixed) (Vazyme, Nanjing, CHN) in a StepOne Plus Real-time PCR System (Applied Biosystems, Singapore city, Singapore). The primer sequences were as follows: *E2F1* (F: 5′-CATCCCAGGAGGTCACTTCTG-3′ and R: 5′-GACAACAGCGGTTCTTGCTC-3′); *ACTB* (F: 5′-CATGTACGTTGCTATCCAGGC-3′ and R: 5′-CTCCTTAATGTCACGCACGAT-3′); *CCNE1* (F: 5′-ACTCAACGTGCAAGCCTCG-3′ and R: 5′-GCTCAAGAAAGTGCTGATCCC-3′); *CDK2* (F: 5′-CCAGGAGTTACTTCTATGCCTGA-3′ and R: 5′-TTCATCCAGGGGAGGTACAAC-3′); *CCND1* (F: 5′-GCTGCGAAGTGGAAACCATC-3′ and R: 5′-CCTCCTTCTGCACACATTTGAA-3′); *CCND3* (F: 5′-TACCCGCCATCCATGATCG-3′ and R: 5′-AGGCAGTCCACTTCAGTGC-3′), *CCNA1* (F: 5′-ACATGGATGAACTAGAGCAGGG-3′ and R: 5′-GAGTGTGCCGGTGTCTACTT-3′). Melting curves were generated to confirm primer specificity.

### Western blot

Cells were collected and lysed with cell lysis buffer (Beyotime, Shanghai, China). Whole-cell extracts were resolved by 10% SDS–PAGE and electrophoretically transferred to polyvinylidene difluoride membranes (Roche Diagnostics, Mannheim, Germany). The blots were cut prior to hybridisation with antibodies during blotting. The membranes were blocked and then incubated with anti-NRF1, anti-β-actin or anti-E2F1 antibodies (Abcam, Cambridge, MA, USA) at 4 °C overnight, followed by incubation with the appropriate horseradish peroxidase-conjugated secondary antibodies (Jackson ImmunoResearch, PA, USA). The chemiluminescence reaction was performed using ECL reagent (Thermo Scientific, IL, USA).

### Clone-forming assay

The cells were seeded (10^3^ cells/well) onto 12-well plates and cultured for 3 days. The cells were fixed with 4% paraformaldehyde for 30 min and stained with crystal violet (Sigma-Aldrich, MO, USA). The cell clones were photographed and counted. Each experimental group was performed in triplicate.

### Cell proliferation assay

The cells were seeded onto 96-well plates at a density of 2 × 10^3^ cells/well and cultured for 96 h. Then, 100 μL of 3-(4,5-dimethylthiazol-2-yl)-2,5-diphenyl-tetrazolium bromide (MTT; Sigma-Aldrich, MO, USA; 5 mg/mL) in PBS was added to each 96-well plate, and the cells were incubated for an additional 4 h. Then, the supernatants were removed and replaced with 100 μL dimethyl sulfoxide to dissolve the formazan crystals. Optical density (OD) was measured at 570 nm wavelength by an ELX-800 Microplate assay reader (Bio-tek, USA). The OD_570_ values indicated changes in cell proliferation.

### Cell cycle analysis

Cells were treated with the serum-free medium for synchronization. To assess the cell cycle distribution, all the above cells were collected and fixed in 70% ethanol overnight. After removal of the ethanol, samples were washed three times with PBS and then incubated with RNase A at 4 °C for 30 min. Next, samples were stained with propidium iodide (50 μg/ml) and evaluated by a Gallios flow cytometer (Beckman). The subsequent analysis was conducted by MultiCycle software.

### Chromatin immunoprecipitation

Cells were fixed with formaldehyde, and sonicated nuclear lysates were processed for immunoprecipitation with NRF1 antibody or normal IgG (Abcam, Cambridge, MA, USA). ChIP DNA fragments were processed for quantitative real-time PCR. The amount of amplified DNA was roughly comparable to that obtained using approximately 2% of the total input chromatin as templates. Primers were designed with *E2F1* promoter binding sites: primer 1 (− 333/− 17), F: 5′-AGAAAGGTCAGTGGGATGCG-3′ and R: 5′-CCAAATCCTTTTTGCCGCGA-3′, which was amplified region of 317-bp; primer 2 (-1291/-869), F: 5′-AGCCTCTGTTTCTTTCATAACCT-3′ and R: 5′-TCGAGACCAGCCTGATCAACA-3′, which was amplified region of 422-bp.

### Plasmid constructs

Genomic DNA was used as the template to construct *E2F1* promoter reporter plasmids. Different truncations of the human *E2F1* promoter were cloned into the pGL3-Basic vector (Promega, WI, USA). Primer sequences for *E2F1* (− 333/− 17) are F: 5′-GCTAGCAGAAAGGTCAGTGGGATGCG-3′ (NheI site is underlined) and R: 5′-AAGCTTCCAAATCCTTTTTGCCGCGA-3′ (HindIII site is underlined); *E2F1* (− 1291/− 869) primer sequences are F: 5′-GCTAGCAGCCTCTGTTTCTTTCATAACCT-3′ (NheI site is underlined) and R: 5′-AAGCTTAGCCTCTGTTTCTTTCATAACCT-3′ (HindIII site is underlined) The NRF1 binding sites in *E2F1* promoter were mutated, respectively. Site-directed mutagenesis of putative NRF1 binding sites was generated using a QuikChange site-directed mutagenesis kit (Stratagene, CA, USA). The expression plasmids for wild-type NRF1 and DN NRF1 (a dominant-negative form) were constructed according to a method described previously [28, 29]. All constructs were verified by sequencing.

### Dual-luciferase reporter assays

Each well of cells was transiently cotransfected with *E2F1* promoter luciferase constructs and pRL-TK (Promega, WI, USA) as an internal control. Cells were lysed and collected to detect luciferase activity by the Dual-Luciferase Reporter Assay System (Promega, WI, USA). The firefly/*Renilla* luciferase activity measurements were recorded according to the manufacturer’s protocol.

### Statistical analysis

The differences in NRF1 expression in tumour and normal tissue were assessed using paired t tests, unpaired Student’s t-tests or Mann–Whitney U tests. Correlations between clinicopathologic features and NRF1 expression were evaluated by the chi-square test. Multivariate survival analysis was performed with Cox regression. Statistical significance was determined by one-way ANOVA, followed by the post hoc Tukey multiple comparison test or two-way ANOVA, followed by Bonferroni's multiple comparisons test. All *P* values reported are from two-sided tests, and the threshold for significance was set at *P* = 0.05. The statistical analyses were performed using STATA version 13.0 (StataCorp, TX, USA).

## Results

### The difference in NRF1 expression in LIHC and normal tissues

**Fig. 1:**
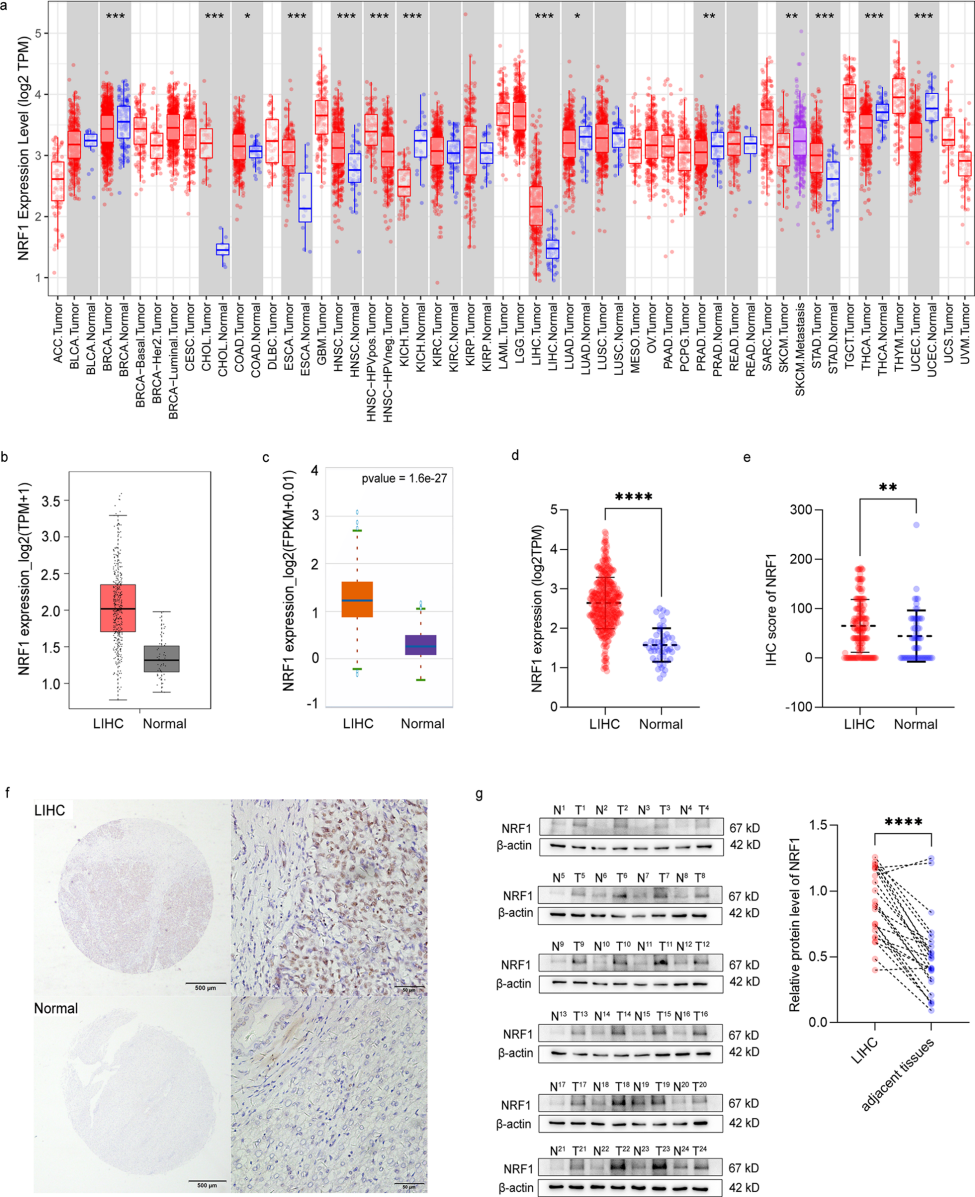
NRF1 expression in cancerous and normal tissues. **a** The expression of IKBIP in different human cancer tissues compared with normal tissues according to the TIMER database. **b**–**d** The level of NRF1 expression in LIHC was obtained from the GEPIA2, ENCORI and TCGA_LIHC databases. **e**, **f** Immunohistochemistry for NRF1 expression in adjacent tissue and LIHC. **g** NRF1 expression in 24 individual LIHC patients was analysed by Western blot and quantified using β-actin as a control. *P < 0.05, **P < 0.01, ***P < 0.001 and ****P < 0.0001 compared with the control

The TIMER database showed that NRF1 mRNA expression was significantly higher in CHOL (bladder urothelial carcinoma), COAD (colon adenocarcinoma), KIRC (kidney renal clear cell carcinoma), KIRP (kidney renal papillary cell carcinoma), and LIHC (liver hepatocellular carcinoma), while it was lower in BRCA (breast invasive carcinoma), LUAD (lung adenocarcinoma), UCEC (uterine corpus endometrial carcinoma), PRAD (prostate adenocarcinoma) and THCA (thyroid carcinoma) than in normal tissues (Fig. 1a). The NRF1 expression in LIHC from GEPIA2 and ENCORI (foldchange = 2.02, *P* = 1.6e−27, FDR = 5.7e−26) datasets were consistent with TIMER (Fig. 1b, c). The RNA-seq profiles from the TCGA_LIHC cohort also showed that NRF1 expression in LIHC was significantly higher than that in the normal group (*P* < 0.0001, Fig. 1d).

Since the public databases contain mRNA expression data, we used IHC to validate in situ protein expression in patient samples. The IHC score significantly differed between LIHC and normal tissues (*P* = 0.0075) (Fig. 1e). Representative images of NRF1 staining are shown in Fig. 1f. Positive NRF1 staining was predominantly localized to the nucleus. NRF1 was negative or weakly stained in normal tissues. Moderate or strong NRF1 staining was found in LIHC. Next, we examined NRF1 protein expression in 24 pairs of LIHC and adjacent noncancerous tissues. The NRF1 expression levels were significantly higher in tumour tissues than in non-tumour tissues (*P* < 0.0001, Fig. 1g).

### NRF1 expression correlated with clinicopathological parameters and poor prognosis

**Fig. 2:**
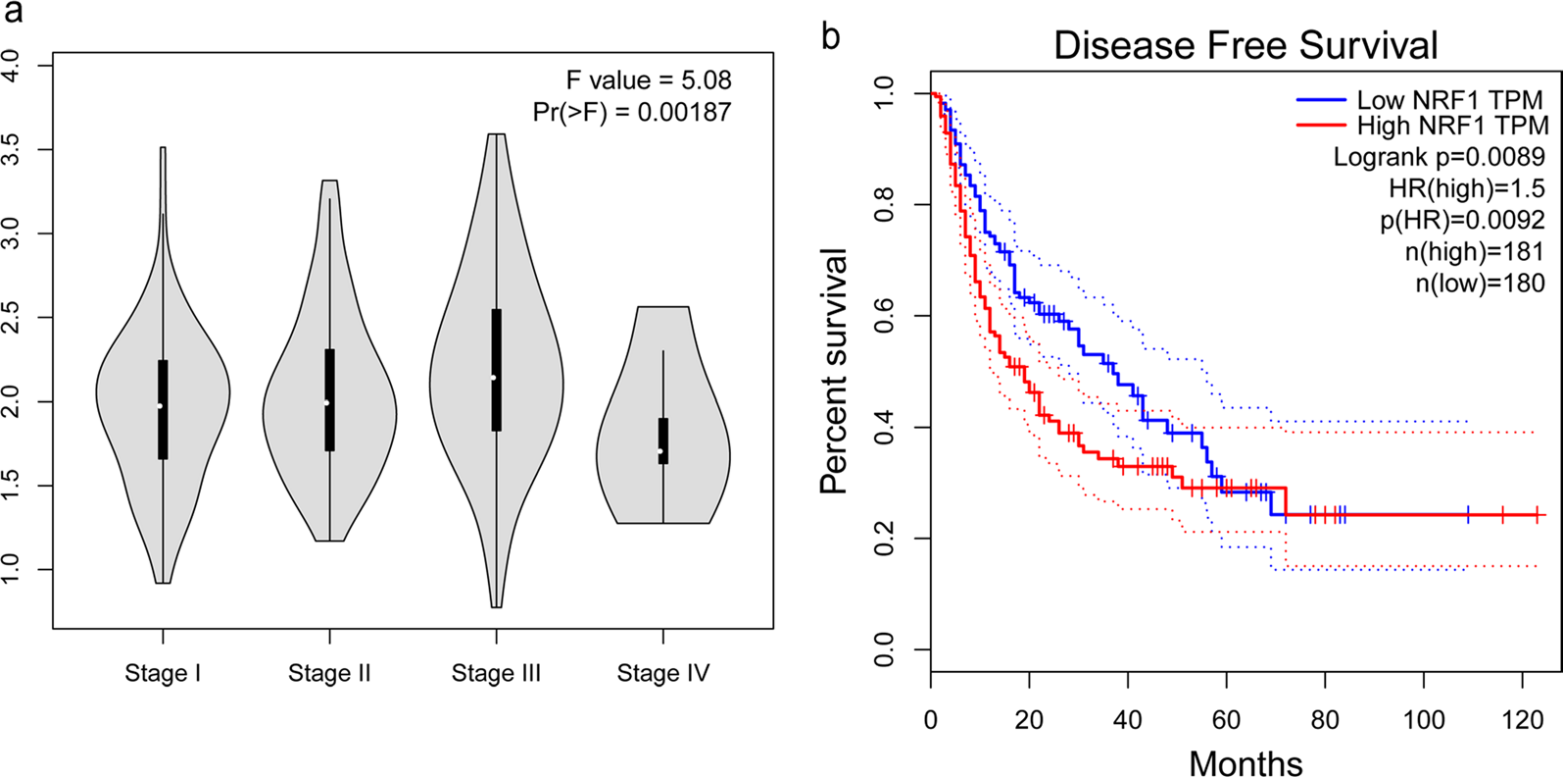
Association between NRF1 expression and clinicopathological parameters. **a** The association of NRF1 expression and different stages of LIHC by using GEPIA2 datasets. **b** Disease-free survival dependent on NRF1 in LIHC patients calculated by the Kaplan–Meier method. Red lines represent patients with higher expression levels of NRF1, and blue lines represent patients with lower expression levels of NRF1 (*P* < 0.001, log-rank test)

The distribution of LIHC patients is shown in Table 1. From our data, NRF1 expression presented a correlation with vascular invasion (*P* = 0.015), TNM stage (*P* = 0.004) and tumour size (*P* = 0.004). In contrast, no correlation (*P* > 0.05) was observed between NRF1 expression and other clinical parameters, including age at diagnosis, differentiation and cirrhosis (Table 2). GEPIA2 datasets were also utilized to analyse the association of NRF1 expression and clinicopathological parameters. As shown in Fig. 2a, there were significant differences between different stages in LIHC patients (*P* < 0.01). Kaplan–Meier survival curves revealed that LIHC patients with high NRF1 expression had significantly poorer disease-free survival (DFS) (*P* < 0.01, HR (hazard ratio) = 1.5, Fig. 2b). The results of Cox regression showed that cirrhosis (*P* = 0.018) and NRF1 expression (*P* = 0.004) correlated with survival of LIHC, and the TNM stage showed a strong tendency towards statistical significance (*P* = 0.052). The relation remained significant after adjustment, and NRF1 (*P* = 0.013; HR_adj_ = 1.87; 95% CI = 1.14–3.06) was found to be an independent prognostic factor (Table 3).

**Table 1:** Characteristics of the populations studied

Characteristic	Detail
N	165
Age	52.64 ± 10.11 years (range 31–79 years)^a^
Sex	125 male, 40 female
Follow-up	44.59 ± 28.96 months (range 1–111 months)^a^

^a^Mean ± SD; range in parentheses

**Table 2:** NRF1 expression and clinical variables in liver hepatocellular carcinoma

Total	NRF1		*P*
	Low	High	
	89 (53.94%)	76 (46.06%)	
Gender			0.212
Female	25 (62.50%)	15 (37.50%)	
Male	64 (51.20%)	61 (48.80%)	
Age			0.804
≤ 50	51 (53.13%)	45 (46.87%)	
> 50	38 (55.07%)	31 (44.93%)	
Grade			0.268
Well and moderate	72 (56.25%)	56 (43.75%)	
poor	17 (45.95%)	20 (54.05%)	
Vascular invasion			0.015*
No	60 (61.86%)	37 (38.14%)	
Yes	29 (42.65%)	39 (57.35%)	
TNM			0.004**
I	41 (64.06%)	23 (35.94%)	
II	38 (56.72%)	29 (43.28%)	
III	10 (29.41%)	24 (70.59%)	
Tumor size			0.004**
≤ 5 cm	62 (63.27%)	36 (36.73%)	
> 5 cm	27 (40.30%)	40 (59.70%)	
Cirrhosis			0.224
No	35 (60.34%)	23 (39.66%)	
Yes	54 (50.47%)	53 (49.53%)	

*P < 0.05, **P < 0.01

**Table 3:** Cox regression analysis of prognostic factors for 5-year survival in hepatocellular carcinoma

	Univariate analysis			Multivariate analysis		
	*P*	HR	95% CI	*P*	HR	95% CI
Gender	0.742	0.91	0.53–1.58			
Male vs Female						
Age	0.227	0.73	0.43–1.22			
< 55 vs ≥ 55						
Grade	0.801	1.08	0.61–1.91			
Well and moderate vs poor						
Vessel invasion	0.208	1.36	0.84–2.21			
No vs yes						
TNM	0.052	1.40	1.00–1.97			
I vs II and III						
Tumor size	0.071	1.56	0.96–2.51			
≤ 5 cm vs > 5 cm						
Cirrhosis	0.018*	1.98	1.13–3.47	0.057	1.74	0.98–3.09
No vs yes						
NRF1	0.004**	2.05	1.26–3.34	0.013*	1.87	1.14–3.06
Low vs high						

*P < 0.05, **P < 0.01

### Effect of NRF1 on cell proliferation

**Fig. 3:**
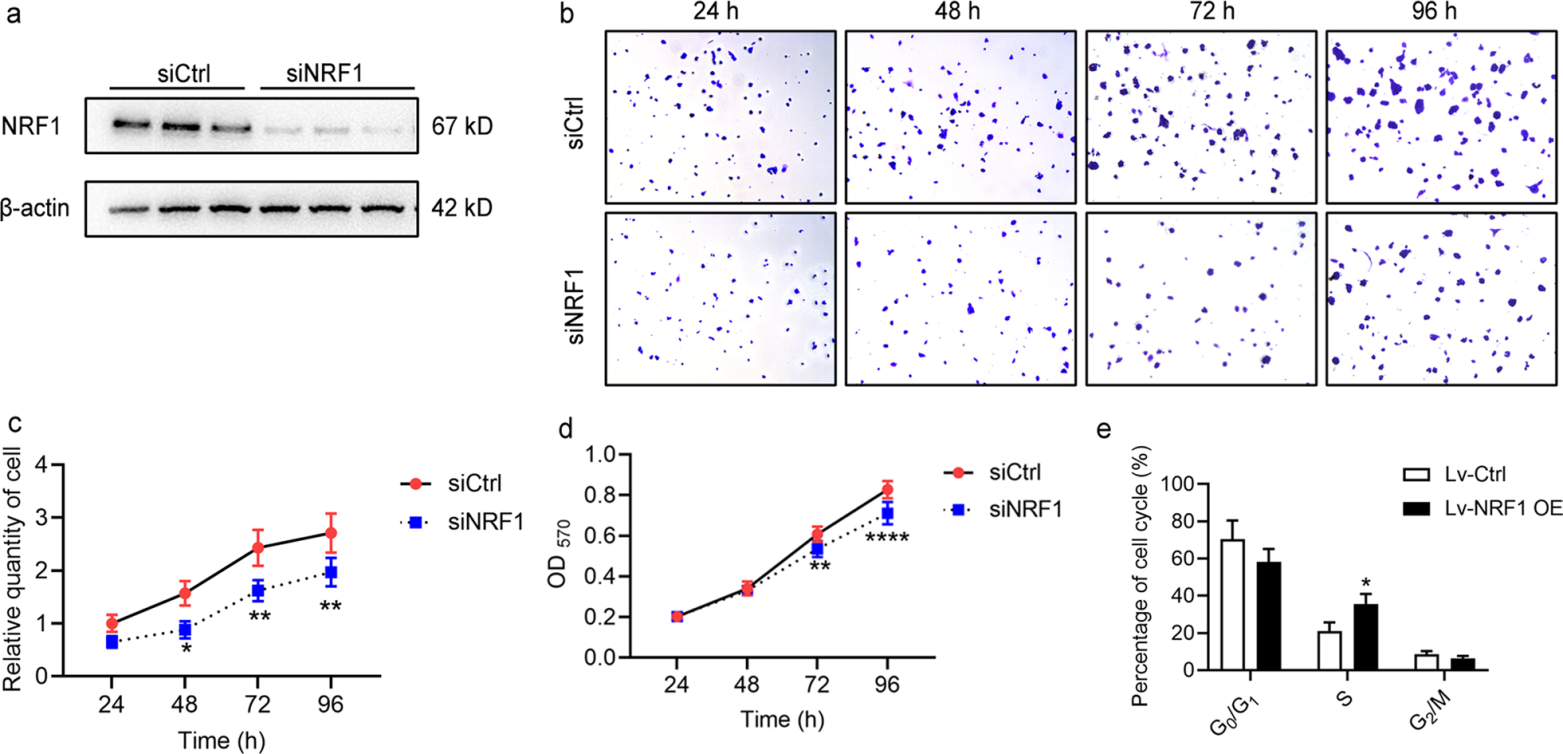
Effect of NRF1 on cell proliferation. **a** HepG2 cells were transfected with siCtrl or siNRF1. NRF1 and β-actin were analysed by Western blot. **b**, **c** Plate colony formation assays and **d** MTT assays were used to measure the impact of NRF1 knockdown on cell clonality and proliferation. **e** The cell cycle distribution of HepG2 cells with stable NRF1 expression was analysed by flow cytometry. *P < 0.05, **P < 0.01 and ****P < 0.0001 compared with the control

Since NRF1 was significantly associated with tumour size, we investigated whether NRF1 expression correlated with liver cancer cell growth. The NRF1-specific siRNA (siNRF1) was used to knockdown the expression of NRF1 (Fig. 3a). The clone formation assay showed that the siNRF1 group had fewer clones than the siCtrl group (Fig. 3b, c). MTT results revealed that fewer cells were found in the siNRF1 group than in the siCtrl group (Fig. 3d). Next, we analysed the proportion of cell populations in each cell cycle phase (Fig. 3e). We used serum starvation-induced cell cycle synchronization to accumulate the cell population prior to G_0_/G_1_. After refeeding with FBS for 24 h, a mass of cells was stimulated to enter the cell cycle and started mitosis simultaneously. The results showed that NRF1 overexpression resulted in a reduction in cells in the G_0_/G_1_ phase and accumulation in the S phase compared with the control, suggesting that NRF1 was involved in the G_1_/S transition (Fig. 3e).

### NRF1 induced E2F1 mRNA expression

**Fig. 4:**
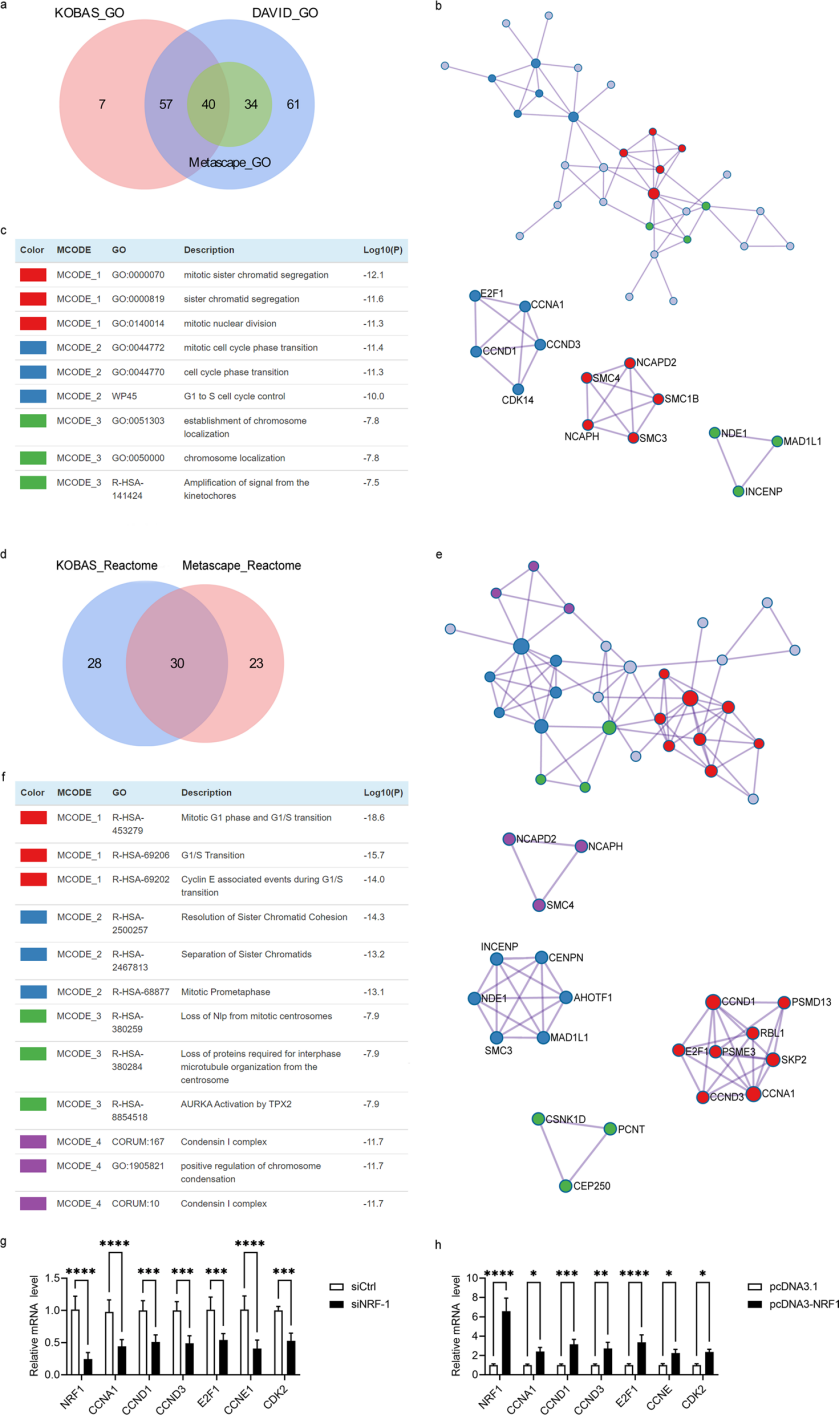
The effect of NRF-1 on E2F1 mRNA. Venn diagram of overlapped genes by GO (**a**) and Reactome analysis (**d**). PPI network constructed from the overlap datasets of GO (**b**) and Reactome (**e**). **c**, **f** The significant module identified from the PPI network using the MCODE method. HepG2 cells were transfected with siNRF1 (**g**) or pcDNA3-NRF1 (**h**). The mRNA levels of NRF1, E2F1, CCND1, CCND3, CCNA1, CCNE1 and CDK2 were detected by real-time PCR using β-actin as a control (n = 3). The data represent means ± SD. *P < 0.05, **P < 0.01 and ****P < 0.0001 compared with the control

ChIP-Seq was performed to detect whether NRF1 target genes were involved in cell growth. All 3984 stringent ChIP-Seq peaks were identified on the Illumina HiSeq analysis platform. DAVID, KOBAS and Metascape were used to identify some NRF1 target genes that showed a correlation with the cell cycle (Additional file 1: Table S1). There were 40 and 30 genes overlapped in GO and Reactome analysis, respectively (Fig. 4a, d, Additional file 1: Table S2).

We next constructed protein–protein interaction network with MCODE applied for module analysis. The most significantly enriched functional modules were those linked to mitotic sister chromatid segregation, cell cycle phase transition (Fig. 4c) and mitotic G_1_ phase and G_1_/S transition (Fig. 4f). Interestingly, the MOCDE results supported that NRF1 overexpression resulted in a reduction in cells in the G_0_/G_1_ phase and accumulation in the S phase (Fig. 3e). The top hub genes related with G_1_/S transition were Cyclin A1 (CCNA1), Cyclin D1 (CCND1), Cyclin D3 (CCND3) and E2F1 (Fig. 4b, e).

As evident from Fig. 4g, there was a striking reduction in CCNA1, CCND1, CCND3 and E2F1 mRNA in siNRF1-transfected cells. Then, we verified that the NRF1 WT construct resulted in a marked increase in CCNA1, CCND1, CCND3 and E2F1 mRNA compared with the pcDNA3.1 control (Fig. 4h). CCND1, CCND3 and CCNA1 belong to the cyclin family, whose function as regulators of CDK kinases. These proteins have been shown to interact with and be involved in the phosphorylation of tumor suppressor protein retinoblastoma (RB). The phosphorylation of RB inhibits heterodimerization with E2F1, and allows E2F1 to be transcriptionally active [30]. The target genes of E2F1 encode proteins that regulate cell cycle progression through the G1/S transition. The Rb/E2F network has a critical role in regulating cell cycle progression and cell fate decisions [31]. Cyclin E1 (CCNE1), which is a target of E2F1, is the limiting factor for G_1_ phase progression and S phase entry [32, 33]. Cyclin E1 activates cyclin-dependent protein kinase 2 (CDK2) shortly before entry of cells into the S phase [34]. Given that cyclin E1 and CDK2 are important regulators of the G_1_/S transition, we questioned whether there was a difference in CCNE1 and CDK2 expression. Consistent with the E2F1 downregulation, attenuation of CCNE1 and CDK2 expression resulted in siNRF1-transfected cells compared with controls (Fig. 4g). In line with our expectations, we observed that CCNE1 and CDK2 were upregulated in the NRF1-overexpressing group (Fig. 4h).

### Identification of NRF1 binding sites in the promoter of the human E2F1 gene

**Fig. 5:**
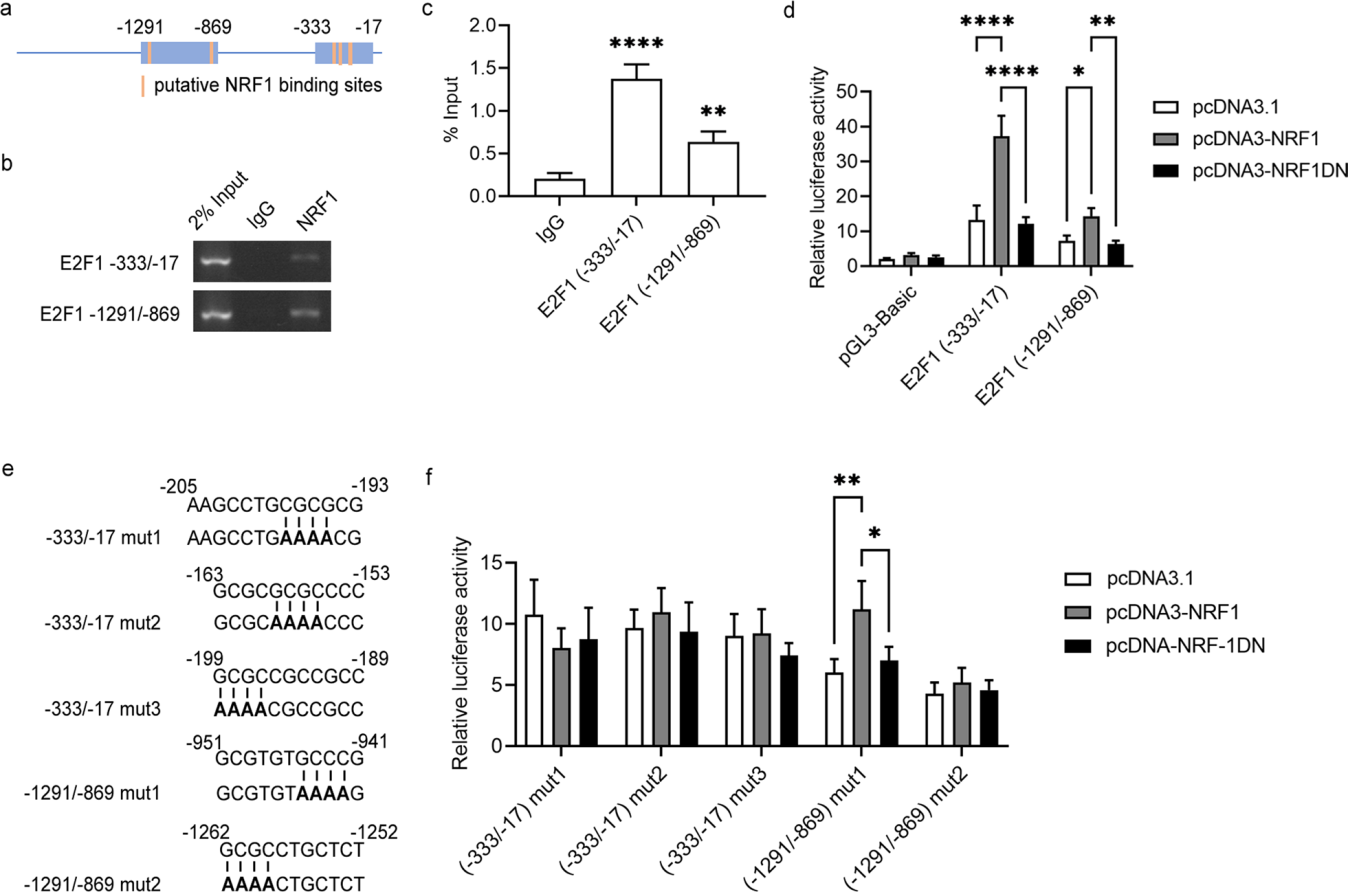
Identification of NRF1 binding sites in the *E2F1* promoter. **a** Schematic presentation of putative NRF1 binding sites on the *E2F1* promoter. **b**, **c** Anti-NRF1 antibody was used for the ChIP assay. Quantification of immunoprecipitated DNA fragments was performed by PCR. **d** The E2F1 constructs (− 333/− 17) or (− 1291/− 869) were cotransfected with pcDNA3-NRF1 or NRF1DN in HepG2 cells. **e, f** Various E2F1 (− 333/− 17) and (− 1291/− 869) constructs harbouring point mutations (mut1 to mut3) were generated and cotransfected with pcDNA3-NRF1 or NRF1DN. The pRL-TK vector was also cotransfected to normalize transfection efficiencies. The luciferase activity was determined by a dual luciferase assay. The results are presented as a luciferase/Renilla ratio. The data represent means ± SD. *P < 0.05, **P < 0.01 and ****P < 0.0001 compared with the control

To identify putative binding sites of NRF1 in the promoter proximal regions of *E2F1*, we performed an in silico search using the open-access database JASPAR (Additional file 1: Table S3). As shown in Fig. 5a, the analysis identified five putative NRF1 binding sites. The in vivo binding of NRF1 to the human *E2F1* promoter was tested by ChIP analysis. Compared with IgG control samples, immunoprecipitated *E2F1* promoter fragments (from − 331 to − 17 and − 1291 to − 869) were significantly enriched using a specific NRF1 antibody (Fig. 5b, c).

We used a luciferase reporter plasmid driven by the human E2F1 promoter region to further evaluate the role of NRF1 in E2F1 transcription. The luciferase activities of the *E2F1* (− 331/− 17) and (− 1291/− 869) constructs were significantly higher than that of the pGL3-Basic construct. Compared with the pcDNA3.1-transfected group, the luciferase activities of *E2F1* promoter constructs were markedly increased in pcDNA3-NRF1-transfected cells. Additionally, no significant changes in E2F1 promoter constructs were detected in the NRF1 DN group (Fig. 5d).

Furthermore, different mutations were detected to identify which nucleotides were essential for *E2F1* transcription by NRF1 (Fig. 5e). Exogenous NRF1 overexpression had no effect on the luciferase activity of *E2F1* (− 333/− 17 mut1 ~ 3) and *E2F1* (− 1291/− 869 mut2). However, a consequent increase in luciferase activity was observed when cells were cotransfected with pcDNA3-NRF1 and E2F1 (− 1291/− 869 mut1). These results illustrated that four functional NRF1 binding sites (from − 205 to − 193, − 163 to − 153, − 199 to − 189 and − 1262 to − 1252) were essential for *E2F1* transcription activity (Fig. 5f).

## Discussion

It has been identified that many classical cancer hallmarks result in altered mitochondrial function [10, 11, 35]. The mitochondrial mass, fission and fusion dynamics, oxidative stress, and mtDNA contribute to tumorigenesis [10, 11, 35].

Mitochondrial homeostasis is intricately regulated by two opposing processes: mitochondrial biogenesis and mitophagy [36]. Mitochondrial biogenesis is a dynamic subcellular process, including import and integrate new proteins and lipids, replicate mtDNA, and fuse and divide in order to adapt to environmental changes [37]. The mitochondrial genome encompasses nuclear DNA (nDNA) genes and mitochondrial DNA (mtDNA). The nDNA contains almost all of the genes for mitochondrial metabolism and biogenesis [38]. NRF1 regulates the transcription of many nuclear-encoded mitochondrial proteins, including the encoding components of the respiratory chain, the mitochondrial protein import machinery, the detoxification response, the heme biosynthetic pathway, and mitochondrial transcription factors [36, 39]. The transcriptional coactivator peroxisome proliferator-activated receptor gamma coactivator-1 alpha (PGC-1α) is a central regulator of mitochondrial biogenesis through interactions with NRF1 [40]. The high levels of PGC-1α often reveal tumor reliance on mitochondrial mass [41]. PGC-1α-dependent mitochondrial biogenesis may contribute to anchorage-independent cancer cell growth, which also supports tumor metastatic potential [42]. Another key activator of mitochondrial biogenesis in cancer is c-Myc. In order to coordinate rapid cancer cell growth, oncogenic c-Myc elevated mitochondrial biogenesis to increase cellular biosynthetic and respiratory capacity [43]. Many MYC binding motif-enriched genes are associated with E2F or NRF1 binding motifs, suggesting that NRF1 may orchestrate both MYC and E2F to regulate common target genes linked to various cancer [44].

Mitophagy is the mitochondrial-specific form of autophagy. The PTEN-induced putative kinase 1 (PINK1)/Parkin-mediated mitophagy plays a key role in mitochondrial quality control [45]. Our previous study has demonstrated that NRF1 has a positive regulatory effect on the transcription of PINK1 and Parkin genes [46]. PINK1 has been identified as a mediator of the PTEN growth-suppressive signaling pathway [47]. The diminished or absent expression of Parkin has been found in a variety of cancers [48]. Therefore, PINK1/Parkin appears to be a novel candidate as tumor suppressor. The promoter of FUN14 domain-containing protein 1 (FUNDC1), which is capable of recruiting the autophagic machinery to mitochondria, contains NRF1-binding sites [49]. Li et al. found that FUNDC1 overexpression significantly increased tumor cell proliferation [50]. In general, FUNDC1 expression was higher in tumor and identified as a detrimental prognostic factor in LIHC. Interestingly, FUNDC1 showed a protective effect on pan-cancer, except LIHC [51]. Similar to autophagy, mitophagy is shown to be both pro- and anti-tumorgenic based on tumor stage and differentiation [52].

The reactive oxygen species (ROS) overproduction in mitochondria promotes cancer development by inducing genomic instability, modifying gene expression, and participating in signaling pathways [53]. NRF1 forms homodimers and regulates cytochrome C oxidase subunit IV (COXIV) and cytochrome c, which are components of respiratory complex [36]. Besides, the mtDNA regulates the 13 most important mitochondrial oxidative phosphorylation genes [54]. Mitochondrial transcription factors, including TFAM, TFB1M and TFB2M, have been reported as target genes of NRF1 [36]. Without all the facts, NRF1 might play a certain role in cancer oxidative stress both directly and indirectly.

Significant efforts on various types of cancers have been made to characterize the extramitochondrial biological processes of NRF1 [28, 29, 55]. NRF1 is essential for lysine-specific demethylase 1 (LSD1) histone modification. The complex of NRF1, LSD1 and oestrogen-receptor related α (ERRα) is required for cell invasion in a matrix metalloprotease 1 (MMP1)-dependent manner [56]. NRF1 also forms an activator complex with egl-9 family hypoxia inducible factor 2 (EglN2) to promote ferridoxin reductase (FDXR) transcription activation. FDXR regulates mitochondrial function and contributes to breast tumorigenesis in vitro and in vivo [57]. SIAH2-NRF1 axis remodels tumor microenvironment for tumor maintenance and progression by regulating tumor mitochondrial function, tumor-associated macrophages (TAMs) polarization and cell death [58].

For decades, mitochondria are symmetrically partitioned to daughter cells during typical cell division [59]. Rehman et al. reported that lung cancer cell lines exhibit an imbalance of mitofusin-2 (Mfn-2) and dynamin-related protein (Drp-1) expression, which mediates mitochondrial fusion and fission [60]. Drp1 and Mfn-2 play a crucial role in controlling cell cycle-associated changes in mitochondrial morphology. Mitra et al. demonstrated a relationship between the mitochondrial form and cell cycle control at the G_1_/S phase [60–63]. In the present study, we demonstrated that NRF1 was correlated with tumour size and promoted cancer cell proliferation in LIHC. Additionally, ChIP-Seq identified some NRF1 target genes that participate in the cell cycle, especially in the G_1_/S phase transition. It is well known that E2F1 was associated with enhanced tumour cell apoptosis or proliferation depending on cell lines and mouse models [64]. E2F1 has contradictory roles in cancer, and its function has been under debate for years [65, 66]. Although the mechanisms have generated some controversy, the core regulatory network of E2F1/Rb that controls the cell cycle in the G_1_/S transition is generally accepted [67]. Previous findings revealed that NRF1 binds to the E2F6 gene promoter [68]. Cam et al. predicted the existence of NRF1 binding sites in E2F target promoters by motif-finding algorithms [69]. Here, we demonstrated that there were four NRF1 binding sites on the *E2F1* promoter that maintained positive transcription in LIHC. Our results confirmed their predictions and suggested that there is an existing link between NRF1 and cell replication. Thus, we hypothesized that the increasing energy demands support cancer rapid proliferation and expansion across the body. Mitochondria is a source of energy for cell metabolism. That resulted in high NRF1 expression for more mitochondrial biogenesis. Beyond bioenergetics support transformation, NRF1 might influence other aspects of mitochondrial biology including fission and fusion dynamics, mitophagy, and oxidative stress regulation to support oncogenesis. Besides, NRF1 up-regulated E2F1 expression transcriptionally, then orchestrated both c-MYC and E2F to regulate their target genes for cancer proliferation.

Several limitations could influence the outcomes of this study. First, our study was retrospective and had a relatively small sample size. DFS analysis is based on RNA-seq data retrieved from public repositories. Hence, the quality and quantity of data can influence the study outcomes, although we verified some outcomes by testing our own clinical samples. Second, racial or ethnic differences were not explained or discussed in our study.

## Conclusions

NRF1 is involved in cancer growth by regulating *E2F1* transcriptionand also a valuable prognostic biomarker for LIHC. Our findings indicated that bioenergetic mitochondrial plasticity and transcriptional networks inevitably should be taken into account when evaluating prognostics and therapeutic options for cancer.

## Supplementary Information

**Additional file 1: Table S1.** The NRF1 downstream genes with a cell cycle association were identified by DAVID, KOBAS and Metascape online databases. **Table S2.** The overlapping NRF1 target genes in GO and Reactome analyses are detailed in the table. **Table S3.** The putative specific loci and the scoring of NRF1 in the promoter proximal regions of E2F1 were analyzed by the open-access database JASPAR.

## Data Availability

The datasets generated and/or analysed during the current study are not publicly available due to limitations of ethical approval involving the patient data and anonymity but are available from the corresponding author on reasonable request.

## Abbreviations

NRF1: Nuclear respiratory factor 1
LIHC: Hepatocellular carcinoma
mtDNA: Mitochondrial DNA
PGC-1α: Peroxisome proliferator-activated receptor gamma coactivator 1α
TCGA: The Cancer Genome Atlas
TIMER: Tumor Immune Estimation Resource
GEPIA: Gene Expression Profiling Interactive Analysis
CHOL: Bladder urothelial carcinoma
COAD: Colon adenocarcinoma
KIRC: Kidney renal clear cell carcinoma
KIRP: Kidney renal papillary cell carcinoma
LIHC: Liver hepatocellular carcinoma
BRCA: Breast invasive carcinoma
LUAD: Lung adenocarcinoma
UCEC: Uterine corpus endometrial carcinoma
PRAD: Prostate adenocarcinoma
THCA: Thyroid carcinoma
siRNA: Small interfering RNA
HR: Hazard ratio
GO: Gene ontology
DFS: Disease-free survival
IHC: Immunohistochemistry
CCNA1: Cyclin A1
CCND1: Cyclin D1
CCND3: Cyclin D3
CCNE1: Cyclin E1
CDK2: Cyclin-dependent protein kinase 2
RT–PCR: Reverse transcriptase-polymerase chain reaction
ChIP: Chromatin immunoprecipitation
LSD1: Lysine-specific demethylase 1
ERRα: Estrogen-receptor related α
EglN2: Egl-9 family hypoxia inducible factor 2
FDXR: Promote ferridoxin reductase
Mfn-2: Mitofusin-2
Drp-1: Dynamin-related protein
