# Supplementary material for: Nuclear respiratory factor 1 promotes the growth of liver hepatocellular carcinoma cells via E2F1 transcriptional activation
Source: BMC Gastroenterol. 2022 Apr 21;22:198. doi: 10.1186/s12876-022-02260-7 (PMC9027447; doi:10.1186/s12876-022-02260-7)
Supplement: Supplementary file 1 — Additional file 1: Table S1. The NRF1 downstream genes with a cell cycle association were identified by DAVID, KOBAS and Metascape online databases. Table S2. The overlapping NRF1 target genes in GO and Reactome analyses are detailed in the table. Table S3. The putative specific loci and the scoring of NRF1 in the promoter proximal regions of E2F1 were analyzed by the open-access database JASPAR. [file 12876_2022_2260_MOESM1_ESM.docx]

**Supplements**

**Table S1 The “cell cycle” enrichment analysis to ChIP-Seq-based NRF1 target genes**

| **Bioinformatic Tool** | **Database** | **ID** | **Term** | **Number** |
| --- | --- | --- | --- | --- |
| **DAVID** | Gene Ontology | 0007049 | Cell cycle | 192 |
|  | KEGG pathway | 04110 | Cell cycle | 12 |
| **KOBAS** | Gene Ontology | 0007049 | Cell cycle | 104 |
|  | Reactome | R-HSA-1640170 | Cell cycle | 58 |
|  | PANTHER | P00013 | Cell cycle | 4 |
|  | KEGG pathway | 04110 | Cell cycle | 15 |
| **Metascape** | Gene Ontology | 0000278 | Mitotic cell cycle | 74 |
|  | Reactome | R-HSA-1640170 | Cell cycle | 46 |
|  | KEGG pathway | 04110 | Cell cycle | 9 |

**Table S2 The overlapped NRF1 putative target genes in cell cycle**

| **Database** | **Gene Name** |
| --- | --- |
| Gene Ontology | ARL3, BABAM1, BOD1, BRSK2, CCNA1, CCND1, CCND3, CDK14, CDKN3, CEP250, CIT, CNTROB, DIS3L2, E2F1, E2F7, FBXL7, INCENP, INO80, KLHL22, KMT5A, LZTS2, MAD1L1, MAU2, NCAPD2, NCAPH, NDE1, PLK5, PPP2R2D, RAD17, REEP3, SIRT2, SMC1B, SMC3, SMC4, SNX9, SON, SPAST, SPDYA, TNKS, USP37 |
| Reactome | AHCTF1, BABAM1, CABLES1, CCNA1, CCND1, CCND3, CDC14A, CENPN, CEP250, CSNK1D, DNA2, E2F1, FBXL7, INCENP, KMT5A, MAD1L1, MAU2, NCAPD2, NCAPH, NDE1, PCNT, PSMD13, PSME3, RAD1, RAD17, RBL1, RNF8, SKP2, SMC3, SMC4 |

**Table S3 The putative binding sites of NRF1**

| **Score** | **Relative score** | **Start** | **End** | **Strand** | **Predicted sequence** |
| --- | --- | --- | --- | --- | --- |
| 12.3355 | 0.929328 | -205 | -195 | + | AAGCCTGCGCG |
| 11.7284 | 0.92183 | -164 | -154 | - | GCGGCGGCGCG |
| 8.92023 | 0.88715 | -199 | -189 | + | GCGCGCGCCCC |
| 8.33556 | 0.87993 | -951 | -941 | - | GCGTGTGCCCG |
| 6.04932 | 0.851695 | -200 | -190 | - | GGGCGCGCGCA |
| 5.73601 | 0.847826 | -163 | -153 | + | GCGCCGCCGCC |
| 5.28917 | 0.842307 | -1262 | -1252 | - | GAGCAGGCGCT |
